# The FPS fingerprint format and chemfp toolkit

**DOI:** 10.1186/1758-2946-5-S1-P36

**Published:** 2013-03-22

**Authors:** Andrew Dalke

**Affiliations:** 1 Andrew Dalke Scientific, 41134, Göteborg, Sweden

## 

During GCC 2010 poster session I presented a draft version of the FPS format for storing dense binary fingerprints. That format is now stable, and supported by RDKit [1], CACTVS [2], and other software. The chemfp package is a set of command-line tools and a Python library for fingerprint generation and high-speed Tanimoto search. It can extract pre-computed fingerprints from an SD tag or use OpenEye's OEChem [3], Open Babel [4], or RDKit to generate fingerprints. Search uses a combination of careful indexing [5], CPU-specific instructions (if available), and OpenMP. Nearest-100 similarity searches of PubChem-sized take less than a second on a laptop, and Butina clustering [6] of 2 million compounds takes about 6 hours on a 15 CPU node. In my poster I present the FPS format and chemfp package, and describe how the memory and performance requirements lead to the internal search architecture.
